# Gonadotropin-Activated Androgen-Dependent and Independent Pathways Regulate Aquaporin Expression during Teleost (*Sparus aurata*) Spermatogenesis

**DOI:** 10.1371/journal.pone.0142512

**Published:** 2015-11-17

**Authors:** Mónica Boj, François Chauvigné, Cinta Zapater, Joan Cerdà

**Affiliations:** 1 IRTA-Institut de Ciències del Mar, Consejo Superior de Investigaciones Científicas (CSIC), 08003, Barcelona, Spain; 2 Department of Biology, Bergen High Technology Centre, University of Bergen, 5020, Bergen, Norway; National Cancer Institute, UNITED STATES

## Abstract

The mediation of fluid homeostasis by multiple classes of aquaporins has been suggested to be essential during spermatogenesis and spermiation. In the marine teleost gilthead seabream (*Sparus aurata*), seven distinct aquaporins, Aqp0a, -1aa, -1ab, -7, -8b, -9b and -10b, are differentially expressed in the somatic and germ cell lineages of the spermiating testis, but the endocrine regulation of these channels during germ cell development is unknown. In this study, we investigated the *in vivo* developmental expression of aquaporins in the seabream testis together with plasma androgen concentrations. We then examined the *in vitro* regulatory effects of recombinant piscine gonadotropins, follicle-stimulating (rFsh) and luteinizing (rLh) hormones, and sex steroids on aquaporin mRNA levels during the spermatogenic cycle. During the resting phase, when plasma levels of androgens were low, the testis exclusively contained proliferating spermatogonia expressing Aqp1ab, whereas Aqp10b and -9b were localized in Sertoli and Leydig cells, respectively. At the onset of spermatogenesis and during spermiation, the increase of androgen plasma levels correlated with the additional appearance of Aqp0a and -7 in Sertoli cells, Aqp0a in spermatogonia and spermatocytes, Aqp1ab, -7 and -10b from spermatogonia to spermatozoa, and Aqp1aa and -8b in spermatids and spermatozoa. Short-term *in vitro* incubation of testis explants indicated that most aquaporins in Sertoli cells and early germ cells were upregulated by rFsh and/or rLh through androgen-dependent pathways, although Aqp1ab in proliferating spermatogonia was also activated by estrogens. However, expression of Aqp9b in Leydig cells, and of Aqp1aa and -7 in spermatocytes and spermatids, was also directly stimulated by rLh. These results reveal a complex gonadotropic control of aquaporin expression during seabream germ cell development, apparently involving both androgen-dependent and independent pathways, which may assure the fine tuning of aquaporin-mediated fluid secretion and absorption mechanisms in the seabream testis.

## Introduction

Spermatogenesis is a coordinated process in which a series of mitotic and meiotic cell divisions of primordial germ cells and differentiating spermatocytes give rise to mature haploid spermatozoa [1, 2]. During this process, drastic morphological and cytological changes occur, and mechanisms involved in rapid fluid transport and efficient cell volume regulation are critical [3]. Thus, during the development of germ cells within the seminiferous epithelium formed by Sertoli cells, fluid secretion is necessary to create a suitable environment for spermatogenesis [4–6]. Changes in the seminiferous tubule fluid also occur as a result of water efflux in round spermatids during their differentiation to spermatozoa (spermiogenesis) [2, 7, 8]. In addition, the control of the fluid composition of the lumen of the efferent ducts and epididymis of mammals is essential for the transport, maturation and concentration of spermatozoa [9–12].

Due to the importance of fluid homeostasis during spermatogenesis, the role of molecular water channels (aquaporins) during these processes has received particular attention [13]. The aquaporins are pore-forming membrane channels that primarily allow the passage of water and other non-charged solutes across biological membranes following an osmotic gradient [14]. In vertebrates, these channel proteins form a superfamily with up to seventeen subfamilies which can be divided into four major groups: the classical water-selective aquaporins (AQP0, -1, -2, -4, -5, -6, -14 and -15), the water and glycerol transporting aquaporins, known as aquaglyceroporins (AQP3, -7, -9, -10 and -13), the AQP8-type aquaammoniaporins, and the unorthodox aquaporins (AQP11 and -12) [15–17]. Numerous studies in mammals have shown that different types of aquaporins are abundant in the testis, including in the interstitial Leydig cells (AQP0, -2, -5, and -9), which are the major source of androgens [18], the Sertoli cells (AQP0, -4, and AQP8-10), the developing germ cells (AQP0, -1, -2, -5, AQP7-9, and -11), and spermatozoa (AQP3, AQP7-9, and -11) [3, 19, 20]. Similarly, multiple aquaporins are found in the different types of epithelial cells of the efferent ducts and epididymis, in which expression can be modulated by steroid hormones such as estrogens and androgens [13]. These findings therefore suggest that aquaporins possibly play important roles controlling the fluid environment needed for germ cell development and the maturation of spermatozoa. However, the specific functions of most aquaporins of the male reproductive tract remain yet unknown.

In teleost fish, testicular fluid transport is also presumably essential during spermatogenesis, as well as during the hydration of the seminal fluid during spermiation, which aids the transport of the sperm through the seminiferous tubules and efferent duct while maintaining the correct osmolality of the seminal plasma [21]. Teleosts harbor a larger repertoire of functionally conserved aquaporin paralogs than mammals as a result of teleost-specific gene duplications [16, 22–24]. A number of studies in evolutionary distant teleosts, such as salmonids, cyprinids, perciforms and flatfishes, have reported the conserved expression of mRNAs encoding different aquaporin paralogs in the testis regardless of their reproduction in freshwater or seawater [22, 23, 25–27]. In the marine teleost gilthead seabream (*Sparus aurata*), Western blot and immunolocalization studies using paralog-specific antibodies indicated the presence of up to seven classes of aquaporins in the testis [28]. In the seabream, Aqp0a and -9b are expressed by Sertoli and Leydig cells, respectively, Aqp1ab, -7, and -10b are in all germ cells from spermatogonia to spermatozoa, and Aqp1aa and -8b are expressed only by haploid spermatids and sperm [28]. These observations indicate a complex pattern of aquaporin localization in the teleost testis as reported for mammals, and suggest that piscine channels may play different roles in water and solute transport during germ cell development.

In order to gain insight into the endocrine control of aquaporin expression in the gilthead seabream testis, in this study we investigated the dynamics of the cell-type-specific expression of Aqp0a, -1aa, -1ab, -7, -8b, -9b and -10b during the spermatogenic cycle. We further investigated their regulation *in vitro* by recombinant piscine gonadotropins, follicle-stimulating (Fsh) and luteinizing (Lh) hormones, as well as by the major steroid hormones involved in teleost spermatogenesis, testosterone (T), the teleost androgen 11-ketotestosterone (11-KT) [29], 17β-estradiol (E2), and the progestin 17α,20β-dihydroxy-4-pregnen-3-one (17,20β-P). Our results reveal an intricate gonadotropic regulation of the different aquaporin paralogs in somatic and germ cells during seabream spermatogenesis which involves steroid-dependent and independent mechanisms.

## Materials and Methods

### Fish and sample collection

Adult gilthead seabream males and females (700 ± 80 g), raised in captivity at the aquaculture facilities of the Institut de Recerca i Tecnologia Agroalimentàries (IRTA) in San Carlos de la Rápita (Tarragona, Spain), were transported to the laboratory and maintained as previously described [28]. At three time points during the natural reproductive cycle, resting period (June-July), initiation of spermatogenesis (September), and spermiation (December), selected males (9, 10 and 3, respectively) were sedated with 500 ppm of phenoxyethanol (Sigma-Aldrich, USA), weighed, and a blood sample taken from the caudal peduncle with a syringe treated with 0.5 M ethylene-diamine-tetraacetic acid (EDTA). Fish were immediately euthanized by decapitation, and the testes removed and weighed in order to determine the gonadosomatic index (GSI; testis weight/body weight x 100). Different biopsies of the testes were deep-frozen in liquid nitrogen and stored at -80°C, used for *in vitro* culture, or processed for histology and immunofluorescence microscopy. Plasma samples were obtained by centrifugation of blood at 10000 x *g* for 10 min at 4°C, and immediately frozen and stored at -80°C. Procedures relating to the care and use of animals and sample collection were carried out in accordance with the protocols approved by the Ethics Committee (EC) of the Institut de Recerca i Tecnologia Agroalimentàries (IRTA, Spain) following the European Union Council Guidelines (86/609/EU). The present study was also specifically approved by IRTA EC.

### Primary antibodies and reagents

The affinity-purified rabbit antisera against seabream Aqp0a, -1aa, -1ab, -7, -8b and -9b have been characterised elsewhere [28, 30, 31]. The antiserum for seabream Aqp10b was raised in rabbits against a synthetic peptide corresponding to amino acid residues 36–50 (AAQVTTSQDKNGQYL; predicted first extracellular domain; GenBank accession no. AAR13054), with the predicted initiation codon (methionine, ATG) designated as residue 1 (Cambridge Research Biochemicals, UK). The serum was further affinity purified against the synthetic peptide. The mouse monoclonal antibodies against rat proliferating cell nuclear antigen (PCNA) and chicken tubulin were purchased from Genetex Inc. (GTX-20029; USA) and Sigma-Aldrich (T9026; USA), respectively. All other reagents and kits were purchased from Sigma-Aldrich unless stated otherwise.

### *In vitro* culture of testicular explants

Testis fragments (10–30 mg) were washed in phosphate-buffered saline (PBS) containing 137 mM NaCl, 2.7 mM KCl, 10 mM Na_2_HPO_4_, and 2 mM KH_2_PO_4_, and transferred to 48-well plates with 300 μl/well of Leibovitz's L-15 culture medium without phenol red (Life Technologies Corp., Spain) supplemented with 10 mM Hepes, 0.5% bovine serum albumin (BSA), 0.4 mg/ml fungizone, and 200 μg/ml penicillin/streptomycin (Life Technologies Corp.). The explants were incubated in quadruplicate or quintuplicate (one explant per well) with 100 ng/ml of European seabass (*Dicentrarchus labrax*) recombinant Fsh or Lh (rFsh and rLh, respectively), or 10 ng/ml of T, E2, 11-KT or 17,20β-P, at 18°C in a temperature-controlled incubator. The methods for the production of seabass rFsh and rLh, and their characterization on seabream gonadotropin receptors, have been published elsewhere [32]. In some experiments, explants were incubated with 5 μM trilostane (TRIL; Selleck Chemicals, USA), an inhibitor of 3β-hydroxysteroid dehydrogenase (Hsd3b), 1 h before the rFsh and rLh treatments. The control explants were incubated with the same concentration of hormone and drug vehicle. After 24h of culture, the medium was collected in two 100-μl aliquots and the testis explants were weighted. Different replicate samples were deep-frozen in liquid nitrogen and stored at -80°C, or processed for immunofluorescence microscopy. For the resting stage, three independent experiments on different pools of three males were carried out, whereas for the spermatogenic stage two individual trials were performed each including a pool of five animals. For the spermiation period, three independent experiments on three different males were carried out.

### Histological analysis

Testis samples from each male collected during the reproductive cycle were fixed in Bouin’s solution (70% picric acid, 20% formaline, 10% acetic acid) for 16 h at room temperature, and dehydrated in increasing ethanol solutions and xylene before embedding in paraplast. Sections of ~8 μm in thickness were attached to UltraStick/UltraFrost Adhesion slides (Electron Microscopy Sciences, USA), deparaffinized with xylene, rehydrated, stained with hematoxilin-eosin, and mounted with Fluka-Eukitt mounting medium. The occurrence of spermatogonia, spermatocytes, spermatids, and spermatozoa, was scored on at least 10 different seminiferous lobules in five different histological sections per fish.

### Steroid determination

The levels T and 11-KT in plasma and culture medium were determined by commercial enzyme immunosorbent assay (EIA; Cayman Chemical Company, USA) as previously described [33]. Free steroids from plasma (100 μl) were extracted in methanol and resuspended in 500 μl of EIA buffer (0.1 M K_2_HPO_4_/KH_2_PO_4_, 1.54 mM sodium azide, 0.4 M NaCl, 1 mM EDTA, and 0.1% BSA, pH 7.4), whereas the culture medium was diluted 1:5 in EIA buffer. All samples were analyzed in duplicate, and for each EIA plate, a separate standard curve was run. Steroid levels in the culture medium were normalized with respect to the weight of the testis explants. The interassay coefficients of variation for T and 11-KT were 2.0 and 4.2%, respectively, whereas the intraassay coefficients of variation for T and 11-KT were 2.5 and 4.4%, respectively.

### RNA extraction and real-time PCR

Total RNA was extracted from the testis using the GenElute^™^ Mammalian Total RNA Miniprep Kit, treated with DNase I, and a 500-ng aliquot reverse transcribed using 0.5 μg oligo (dT)_17_, 1 mM dNTPs, 40 IU RNAse inhibitor, and 10 IU SuperScript II (Life technologies Corp.) for 1.5 h at 42°C. Real-time quantitative RT-PCR (qRT-PCR) was carried out using 5 μl of SYBR Green qPCR master mix (Life Technologies Corp.), 1 μl of diluted cDNA (1:25 for *aqp0a* and *-8b*, 1:50 for *aqp7*, *-9b*, and *-10b*, and 1:150 for *aqp1aa*, and *-1ab*), and 0.5 μM of each primer (S1 Table). The reference gene was 18s ribosomal protein (*rps18*) and the cDNA was diluted 1:150 (S1 Table). Each sample was assayed in duplicate on 384-well plates using the ABI PRISM 7900HT sequence detection system (Applied Biosystems, Life technologies Corp.). The amplification protocol was an initial denaturation and activation step at 50°C for 2 min and 95°C for 10 min, followed by 40 cycles of 95°C for 15 s and 63°C for 1 min. After the amplification phase, a temperature-determining dissociation step was carried out at 95°C for 15 s, 60°C for 15 s, and 95°C for 15 s. To estimate the primer efficiencies, a standard curve was generated for each primer pair from 10-fold serial dilutions (from 1 to 0.00001) of a pool of mixed testis cDNA templates. All calibration curves exhibited correlation coefficients >0.99, and the corresponding quantitative qRT-PCR efficiencies ranged from 1.9 to 2.1 (S1 Table). Changes in gene expression in testicular explants cultured *in vitro* were determined as fold-changes with respect to untreated explants at time zero using the 2^-ΔΔCt^ method [34].

### Immunofluorescence microscopy

Testis explants were fixed in 4% paraformaldehyde (PFA) for 6 h, washed, dehydrated, and embedded in paraffin as previously described [28]. Sections were blocked in 5% goat serum and 0.1% BSA in PBS with 0.1% Tween-20 (PBST) for 1 h, and incubated with PBS containing 0.2%Triton X-100 for 10 min at room temperature (Aqp1aa, -8b and -9b antibodies), or PBS containing 0.2% SDS for 10 min at room temperature (for Aqp1ab, -0a, -7 and -10b antibodies). Incubation with the antibodies was performed overnight at 4°C in PBS at 1:400 (for Aqp0a, -1aa, -8b, -9b, and -7 antisera) or 1:200 (for Aqp1ab and -10b antisera). A set of slides was incubated with the antibodies preadsorbed with the respective immunizing peptides as negative controls. After washing, sections were probed with a sheep Cy3-coupled anti-rabbit IgG secondary antibody (1:600; Sigma-Alrich, C2306) for 1 h at room temperature. The nuclei were counterstained with 4’,6-diamidino-2-phenylindole (DAPI) at 1:3000 in PBS for 3 min, and mounted with fluoromount aqueous anti-fading medium. Sections were examined and photographed with a Zeiss Axio Imager Z1/ApoTome fluorescence microscope (Carl Zeiss Corp.). Images from sections at different spermatogenic stage or after hormone treatments were taken with the same fluorescence intensity and exposure than those used for the controls.

### PCNA assay

Histological sections were incubated in boiling citrate buffer (10 mM sodium citrate, pH 6) for 20 min. The sections were subsequently probed with the PCNA antibody (1:300) overnight at 4°C, and then with a goat Alexa Fluor 488-coupled anti-rabbit IgG secondary antibody (1:600; Life Technologies Corp., A-11008). Negative controls were incubated with the secondary antibody only. The percentage of proliferative germ cells (spermatogonia only) per tubule was quantified by manually counting the PCNA-positive cells in 5 different testicular regions per replicate treatment under a fluorescence microscope.

### Protein extraction and immunoprecipitation

Replicate testis fragments were dissociated with a glass dounce homogenizer in ice-cold lysis buffer containing 150 mM NaCl, 50 mM Tris-HCl, pH 7.4, 1% Triton X-100, 0.25% sodium deoxycholate, 1 mM EDTA, 1 mM Pefablock, EDTA-free protease inhibitors (Roche, Spain), 1 mM Na_3_VO_4_, and 1 mM NaF, and centrifuged at 14000 x *g* for 5 min at 4°C. One aliquot of supernatant was removed to determine the protein concentration with the Bio-Rad Protein Assay kit (Bio-Rad Laboratories Inc., USA), and the rest of the supernatant was mixed with 2x Laemmli sample buffer, frozen in liquid nitrogen, and stored at -80°C. For the spermatogenic period, testis explants from two experiments on two different pools of fish were combined for each treatment before homogenization.

Direct immunoprecipitation (IP) using the seabream Aqp1ab antibody was carried out to concentrate Aqp1ab protein in the resting stage. For this, testis explants from three experiments were pooled for each treatment, homogenized in cold IP buffer (50 mg/ml) containing 150 mM NaCl, 50 mM Tris-HCl pH 7.4, 1% Triton X-100, 0.5% sodium deoxycholate, 5 mM EDTA, 5 mM ethylene glycol tetraacetic acid (EGTA), 1 mM Pefablock, 0.1% SDS, and EDTA-free protease inhibitors, and mixed with the same amount of antibody-coupled Pure Proteome^™^ Protein G Magnetic Beads (Merck Millipore, Spain) following the manufacturer’s instructions.

### Western blotting

Total (20–60 μg) and immunoprecipitated proteins were denatured at 95°C for 10 min, electrophoresed in 12% SDS-PAGE, and blotted onto nitrocellulose membranes as previously described [28]. The membranes were blocked with 5% nonfat dry milk diluted in TBST (20 mM Tris, 140 mM NaCl, 0.1% Tween; pH 8) for 1 h at room temperature, and subsequently incubated overnight at 4°C with the different aquaporin and alpha-tubulin antibodies diluted in TBST with 1% nonfat dry milk (1:400 for anti-Aqp1aa, -7, and -10b; 1:500 for Aqp0a and -9b; 1:800 for Aqp1ab and -8b; and 1:4000 for anti-alpha-tubulin). Horseradish peroxidase (HRP)-coupled anti-rabbit or mouse IgG secondary antibodies (1:5000; Santa Cruz Biotechnology Inc., sc-2004 and sc-2005, respectively) were applied for 1 h at room temperature, and reactive bands were detected using the Immobilon^™^ Western chemiluminescent HRP substrate (Merck Millipore). For semi-quantitative determination of aquaporin abundance in explants at the spermatogenic and spermiation stages, the intensity of the immunoreactive bands was determined by densitometry using the Quantity-One software (Bio-Rad Laboratories Inc.) and normalized to that of alpha-tubulin. For explants at the resting stage, the intensities of the Aqp1ab-immunoprecipitated bands were normalized to that of the IgG heavy chain. Data were represented as fold-changes with respect to untreated explants at time zero.

### Statistics

Data are the mean ± S.E.M. and were statistically analyzed by the one- or two-way ANOVA, after log-transformation of the data when needed, followed by the Duncan’s multiple range test. Statistical analyses were carried out using the Statgraphics Plus 4.1 software (Statistical Graphics Corp., USA). A *P* value < 0.05 was considered statistically significant.

## Results

### Germ cell development and androgen plasma levels during the seabream spermatogenic cycle

**Fig 1:**
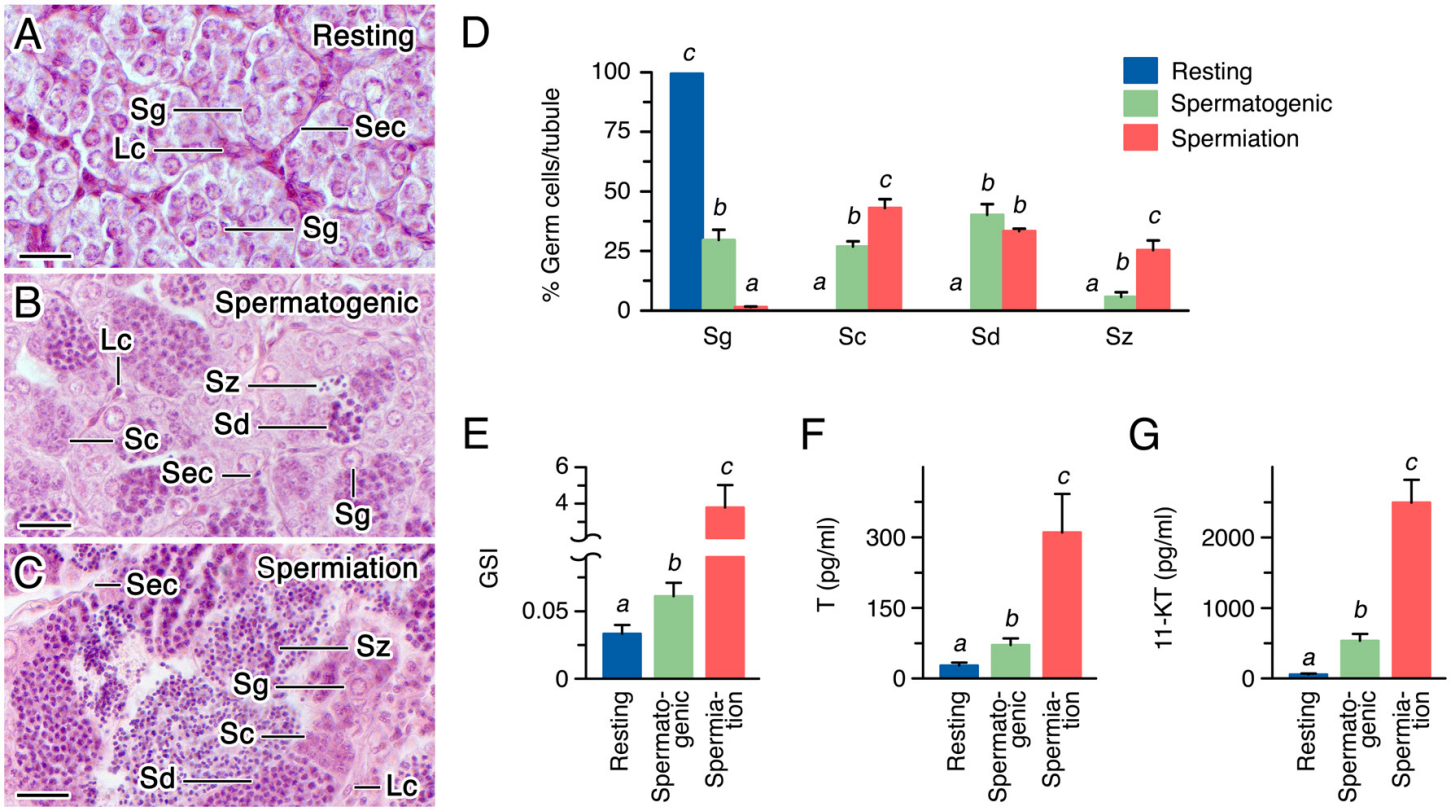
Characterization of the gilthead seabream spermatogenesis stages investigated in this study. (A-C) Representative histological sections of testis at the resting (A), spermatogenic (B) and spermiation (C) stages stained with hematoxylin and eosin. Scale bars, 20 μm. (D) Percentage of germ cells per seminiferous lobule in each of the three spermatogenic stages. In A-D, Sec, Sertoli cells; Lc, Leydig cells; Sg, spermatogonia; Sc, spermatocytes; Sd, spermatids; Sz, spermatozoa. (E-G) GSI (E), and T (F) and 11-KT (G) plasma levels, of males showing testes at the resting, spermatogenic and spermiation stages. In D-G, bars (mean ± S.E.M.; n = 6, 8 or 3 fish, for the resting, spermatogenic and spermiation stages, respectively) with different superscript letters are significantly different (P < 0.05).

The spermatogenic stages investigated in the present study, resting, spermatogenic and spermiation, were characterized histologically to establish the progression of germ cell development. At the resting stage, the testis was formed by small seminiferous tubules, which did not show a continuous lumen and predominantly contained spermatogenic cysts formed by groups of spermatogonia only (Fig 1A and 1D). In the spermatogenic testis meiosis was initiated, and accordingly the tubules contained a decreased relative number of spermatogonia and increased number of spermatocytes and spermatids, together with some spermatozoa within the lumen of the cysts which starts to open (Fig 1B and 1D). In spermiating males, the lumen of the testicular tubules prominently enlarged and was filled by a high number of spermatozoa, whereas spermatocytes and spermatids, and a few spermatogonia, remained in the periphery of the lobules (Fig 1C and 1D). The progression of spermatogenesis from the resting to the spermiating stage was associated with an increase of ~100-fold in the GSI (Fig 1E), and with a surge of the plasma levels of T and 11-KT of ~11- and ~44-fold, respectively (Fig 1F and 1G).

### Cellular localization of aquaporins during spermatogenesis *in vivo*

**Fig 2:**
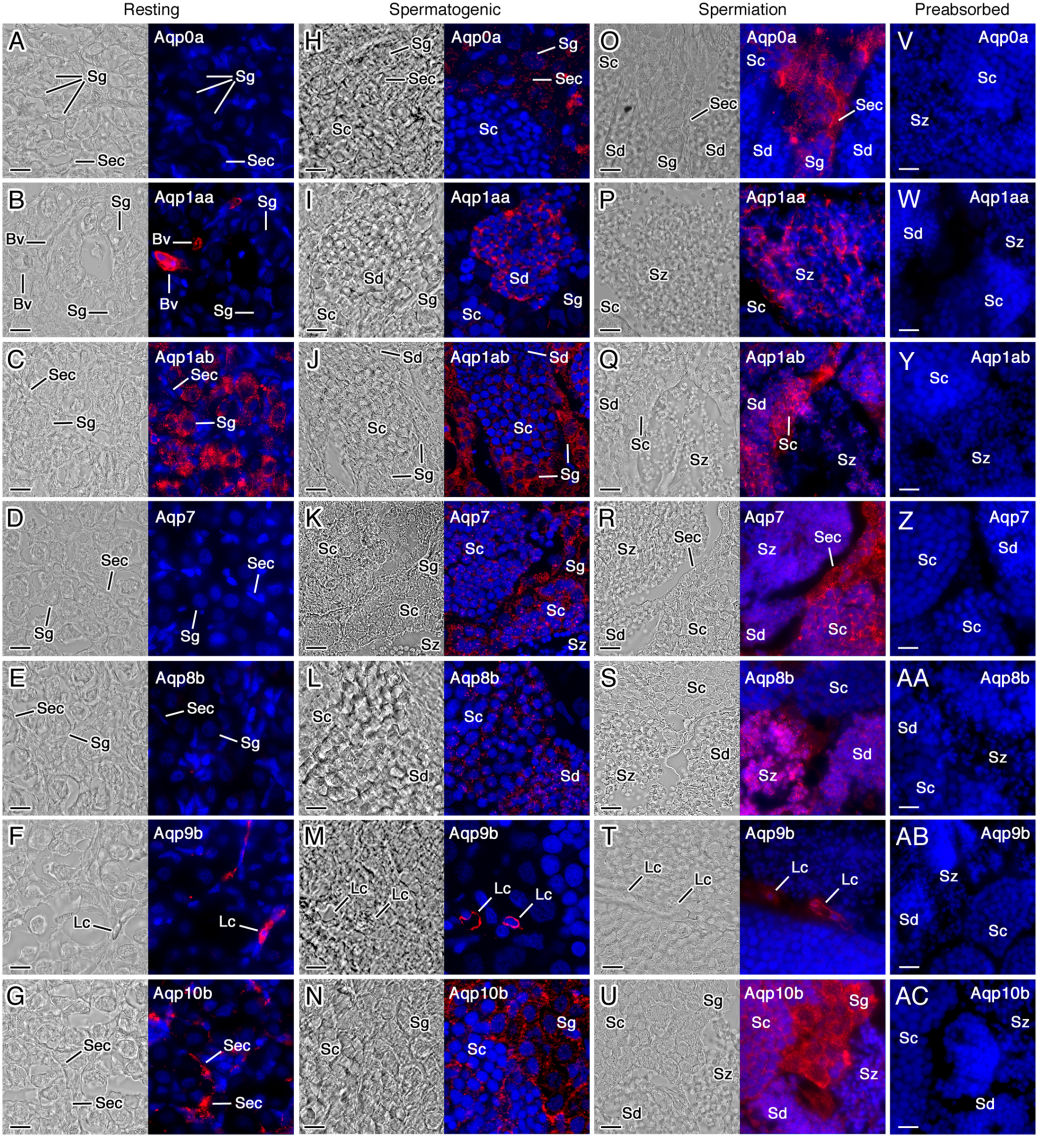
Cellular localization of aquaporins in the gilthead seabream testis at different spermatogenesis stages detected by immunofluorescence microscopy. Representative bright field (left panels) and immunofluorescence (right panels) microscopy images of Aqp0a, -1aa, -1ab, -7, -8b, -9b and -10b localization in testes at the resting, spermatogenic and spermiation stages as indicated. Sections were labeled with affinity-purified paralog-specific rabbit polyclonal antibodies. The reactions were visualized with Cy3-coupled anti-rabbit IgG secondary antibodies (red) and the nuclei were counterstained with DAPI (blue). Control sections at the spermiation stage (V-AC) were incubated with preabsorbed antisera. The same results were obtained on control sections at the resting or spermatogenic stages (data not shown). Sec, Sertoli cell; Lc, Leydig cell; Sg, spermatogonia; Sc, spermatocyte; Sd, spermatid; Sz, spermatozoa. Scale bars, 10 μm.

The cell type-specific expression of Aqp0a, -1aa, -1ab, -7, -8b, -9b and -10b in the seabream testis during the three spermatogenic stages was determined by immunofluorescence microscopy using paralog-specific antibodies (Fig 2). During the resting period, Aqp0a, -7 and -8b were not detected (Fig 2A, 2D and 2E), whereas Aqp1aa was restricted to the endothelia of blood vessels (Fig 2B) and Aqp1ab appeared to be distributed mostly in the cytoplasm of spermatogonia (Fig 2C). Aqp9b was detected exclusively in the plasma membrane and cytoplasm of isolated or grouped interstitial cells, which were classified as Leydig cells based on their distribution and nuclear morphology (Fig 2F). In contrast, Aqp10b was immunolocalized in Sertoli cells sustaining the spermatogonial cysts (Fig 2G).

With the progression of germ cell development during the spermatogenic stage, Sertoli cells started to weakly express Aqp0a (Fig 2H) and -7 (Fig 2K), in addition to Aqp10b (Fig 2N), whereas Aqp9b remained in Leydig cells (Fig 2M). The entry into meiosis of germ cells during spermatogenesis appeared to be associated with the translational activation of Aqp0a (Fig 2H), -7 (Fig 2K) and -10b (Fig 2N) in spermatogonia, together with that of Aqp1ab, which persisted from the resting stage (Fig 2J). However, only Aqp1ab and -7 polypeptides continued to be clearly expressed in spermatocytes and spermatids at this stage (Fig 2J and 2K). The expression of Aqp1aa and -8b was activated late during the spermatogenic stage since specific immunostaining for these channels was only observed in spermatocytes and spermatids (Fig 2I and 2L).

During the spermiation period, intense Aqp0a signals further appeared in spermatocytes as well as in spermatogonia and Sertoli cells (Fig 2O). The expression of Aqp1aa (Fig 2P), -1ab (Fig 2Q), -7 (Fig 2R) and -8b (Fig 2S) in germ cells, and of Aqp7 and -9b in Sertoli (Fig 2R) and Leydig (Fig 2T) cells, respectively, remained unchanged with respect to that observed in the spermatogenic stage, but in general with more intense fluorescence signals. In contrast, Aqp10b was found for the first time abundantly expressed in spermatocytes and spermatids, in addition to spermatogonia and Sertoli cells, during spermiation (Fig 2U). At this stage, spermatozoa accumulating in the tubule lumen were immunostained only for Aqp1aa (Fig 2P), -1ab (Fig 2Q), -7 (Fig 2R), -8b (Fig 2S) and -10b (Fig 2U).

The preincubation of the antibodies with the corresponding immunizing peptides completely abolished the immunoreactions in all spermatogenic stages (Fig 2V–2AC), demonstrating the specificity of the staining and thus supporting the differential spatial and temporal expression of aquaporins during seabream spermatogenesis *in vivo*.

### Transcriptional regulation of testicular aquaporins by gonadotropins and steroids *in vitro*

**Fig 3:**
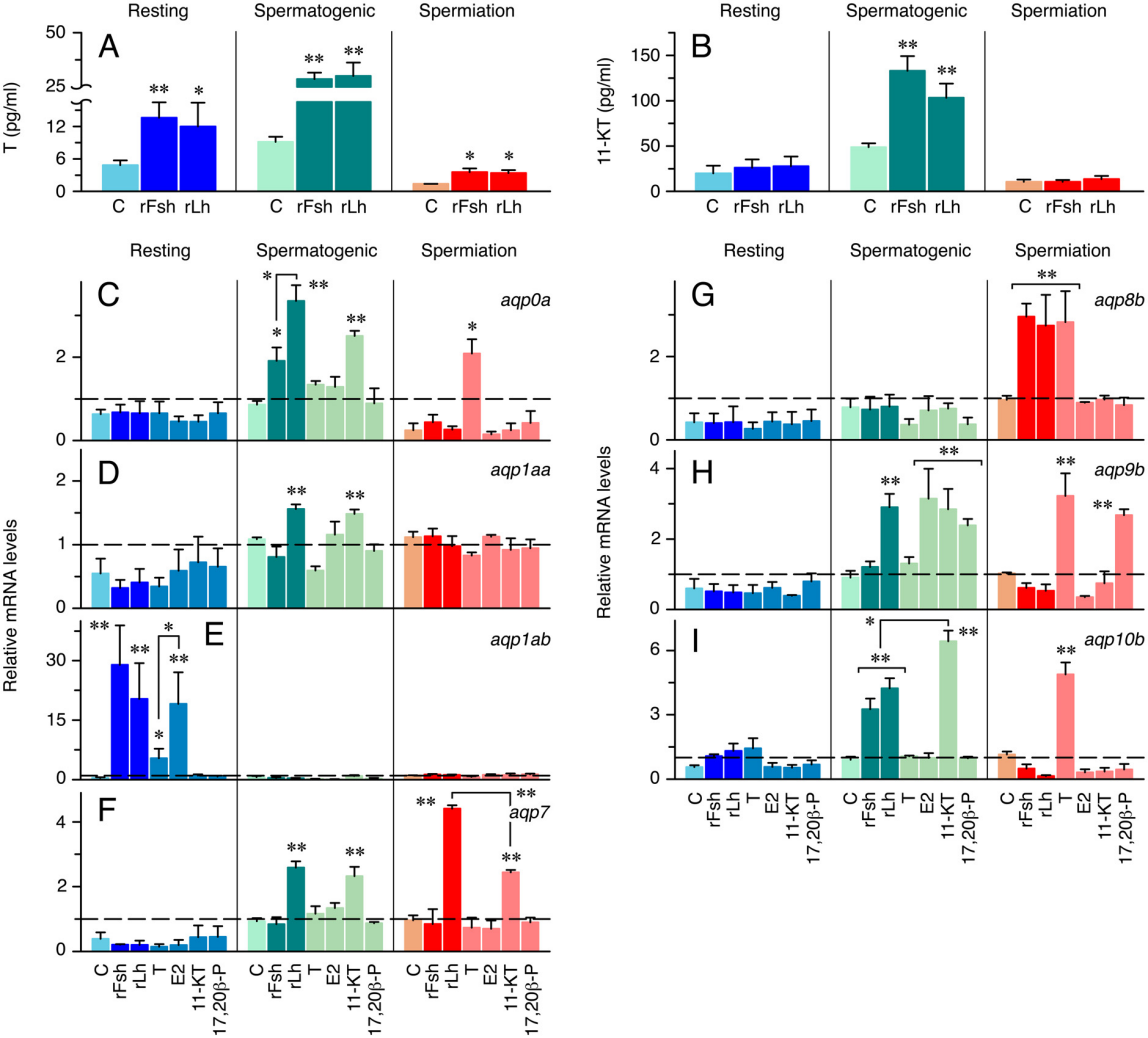
Hormone control of steroid production and aquaporin gene expression in the seabream testis *in vitro*. (A-B) Amounts of T (A) and 11-KT (B) released into the incubation media by explants at the resting, spermatogenic and spermiation stages after 24 h exposure to 100 ng/ml of rFsh or rLh, or hormone vehicle (control; C). (C-I) Effect of 100 ng/ml of rFsh or rLh, or of 10 ng/ml of steroid hormones (T, E2, 11-KT or 17,20β-P) on *aqp0a* (C), *aqp1aa* (D), *aqp1ab* (E), *aqp7* (F), *aqp8b* (G), *aqp9b* (H) and *aqp10b* (I) expression during spermatogenesis. Histograms represent relative mean expression values normalized to *rps18* gene expression, and expressed as fold-changes with respect to untreated explants at time zero. Dashed line at mRNA level 1 indicates no change with respect time zero. In A-I, values (mean ± S.E.M.) from the resting and spermiation stage are from three independent experiments on three different pools of males or individual males, respectively, each with two replicates per condition. For the spermatogenic stage, values (mean ± S.E.M.) represent compiled data from two experiments on two different pools of males, each with three replicates per hormone treatment. *, P < 0.05; **, P < 0.01, with respect the control group, or as indicated in brackets.

To investigate the gonadotropic control of aquaporin expression in the seabream testis, rFsh and rLh from another perciform teleost, the European seabass, were used as gonadotropin sources, since previous studies have shown that these hormones are able to activate the gilthead seabream Fsh and Lh receptors [32]. Initial experiments were designed to establish the potency of rFsh and rLh at inducing androgen release in the culture medium by testicular explants incubated *in vitro* for 24 h. In explans at the resting period, both rFsh and rLh stimulated the production of T by ~3-fold above basal levels (Fig 3A), but not that of 11-KT (Fig 3B). At the spermatogenic stage, the basal androgen production was higher than in the previous period, but in this case rFsh and rLh induced a similar increase of both T and 11-KT by ~3-fold (Fig 3A and 3B). Finally, at the spermiation phase the steroidogenic capability of explants appeared to decrease, since the basal production of T and 11-KT was lower than during the spermatogenic period (Fig 3A and 3B). At this stage, the rFsh and rLh also induced a slightly lower increase of T of ~2.5-fold, but they were completely ineffective at stimulating 11-KT secretion (Fig 3A and 3B).

The gonadotropic regulation of the expression of *aqp0a*, *-1aa*, *-1ab*, *-7*, *-8b*, *-9b* and *-10b* was investigated by qRT-PCR using paralog-specific oligonucleotide primers in testes explants at the resting, spermatogenic and spermiation periods, incubated in the presence of rFsh and rLh, as well as of different steroid hormones such as T, E2, 11-KT and 17,20β-P, for up to 24 h. Before the expression levels were evaluated, however, we investigated whether these hormone treatments could stimulate the proliferation of germ cells *in vitro* using a PCNA antibody (S1 Fig). During the resting period, both rFsh and rLh, as well as E2, significantly but moderately stimulated the percentage of proliferative spermatogonia in the testis explants (~2-fold above control levels), whereas T was the most potent hormone at incrementing cell proliferation (~4-fold increase with respect the controls). In contrast, at the spermatogenic stage only T elicited a small increase of proliferative spermatogonia (~2.5-fold with respect the controls), while at the spermiation stage both rFsh and T induced a considerable increase of proliferative cells (~3- and ~5-fold above basal levels, respectively).

The results of the qRT-PCR experiments showed a differential regulation of aquaporin transcripts by gonadotropins and steroids which changed depending on the developmental stage of the testes. Thus, *aqp0a* expression was not affected by the hormones at the resting stage, while at the spermatogenic stage it was upregulated by rFsh and particularly by rLh, as well as by 11-KT (Fig 3C). In contrast, during spermiation *aqp0a* was activated only by T (Fig 3C). Testicular *aqp1aa* (Fig 3D) and *-7* (Fig 3F) transcripts were not affected during the resting period, whereas both mRNAs were stimulated by rLh and 11-KT during spermatogenesis, and in the case of *aqp7*, also at the spermiation stage. *aqp1ab* was the only transcript strongly up-regulated by rFsh, rLh, T, and E2 at the resting period (Fig 3E). However, while the stimulation of *aqp1ab* by rFsh and rLh was of similar magnitude, that of E2 was significantly more potent than that elicited by T (Fig 3E). Interestingly, the levels of *aqp1ab* were no longer regulated by hormones at the spermatogenic or spermiation stages. *aqp8b* was only activated by gonadotropins and T at the spermiation stage (Fig 3G), whereas the levels of the Leydig cell-specific *aqp9b* were stimulated by rLh, E2, 11-KT and 17,20β-P during spermatogenesis, but only by T and 17,20β-P during spermiation (Fig 3H). Finally, expression of *aqp10b* was stimulated by rFsh, rLh and 11-KT during spermatogenesis, but it was activated only by T during spermiation (Fig 3I). These data therefore indicated that *aqp1ab* and *-8b* are specifically regulated during spermatogonia proliferation and spermiation, respectively, whereas the expression of *aqp0a*, *-1aa*, *-7*, *-9b* and *-10b* is mainly controlled during spermatogenesis.

### Androgen-dependent and -independent actions of Fsh and Lh on aquaporin gene expression *in vitro*

**Fig 4:**
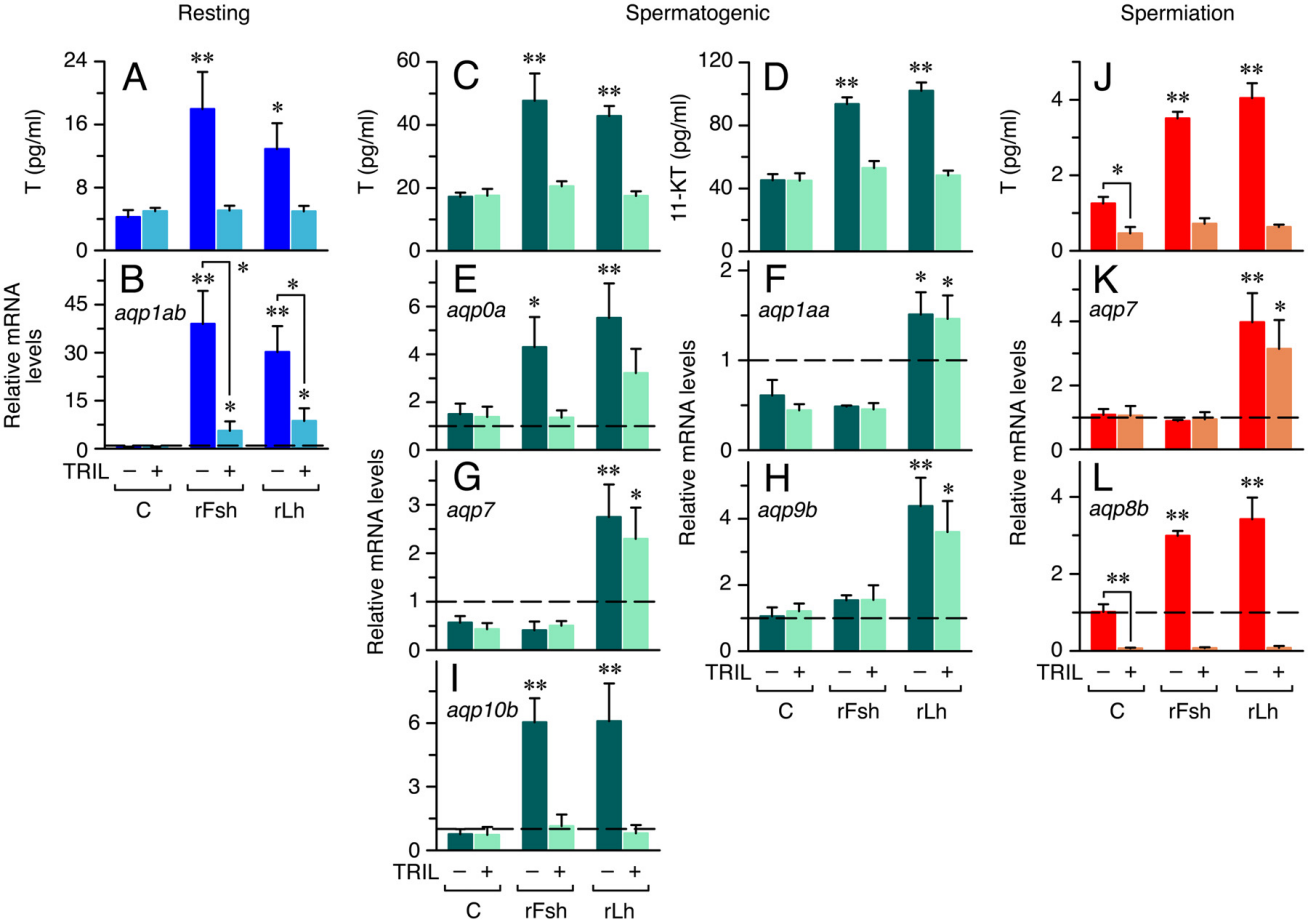
Effect of the steroidogenesis inhibitor TRIL on gonadotropin-induced androgen production and aquaporin transcriptional regulation *in vitro*. (A, C-D, J) Amounts of T (A, C, J) and 11-KT (D) released into the incubation media by explants at the resting, spermatogenic and spermiation stages after 24 h exposure to 100 ng/ml rFsh or rLh, or hormone vehicle (control; C), in the presence or absence of 5 μM TRIL. (B, E-H, K-L) Effect of TRIL on rFsh and rLh-induced aquaporin gene expression during spermatogenesis. Data are represented as in Fig 3. Dashed line at mRNA level 1 indicates no change with respect time zero. Values (mean ± S.E.M.) represent compiled data from two experiments on two different pools of males or individual males, each with three replicates per treatment. *, P < 0.05; **, P < 0.01, with respect the control group not treated with TRIL, or as indicated in brackets.

To determine whether the effects of gonadotropins on aquaporin gene expression were dependent on steroid production, the transcript levels of the rFsh- and rLh-regulated genes observed earlier were re-evaluated in explants cultured *in vitro* in the presence of gonadotropins and the steroidogenesis inhibitor TRIL by qRT-PCR (Fig 4). As expected, TRIL abolished the rFsh and rLh stimulation of T production at the resting period (Fig 4A), as well as the rFsh and rLh induction of *aqp1ab* expression (Fig 4B), indicating that the effects of both gonadotropins were mediated by T. During the spermatogenic stage, TRIL also reduced gonadotropin-stimulated T (Fig 4C) and 11-KT (Fig 4D) secretion to basal levels, but the inhibitor had different effects on aquaporin expression. While TRIL completely prevented the rFsh and rLh activation of *aqp10b* (Fig 4I) and the rFsh-induced *aqp0a* (Fig 4E), and partially blocked the accumulation of *aqp0a* caused by rLh (Fig 4E), it did not affect the stimulation of *aqp1aa* (Fig 4F) and *-9b* (Fig 4H) by rLh. At the spermiation stage, the presence of TRIL reduced the basal and gonadotropin-stimulated production of T (Fig 4J). However, the rLh-induced *aqp7* upregulation was not affected by TRIL (Fig 4K), whereas the rFsh and rLh activation of *aqp8b*, as well as its basal expression, were completely inhibited (Fig 4L). These and previous data suggested that aquaporin expression in the seabream testis is regulated by steroid-dependent and independent mechanisms.

### Translational control of testicular aquaporins *in vitro*

To investigate whether the endocrine regulation of aquaporin mRNA expression in the testis correlates with the corresponding proteins, the explants previously stimulated with gonadotropins and steroids were further analyzed by Western blot. Although not quantified, the explants were also examined by immunofluorescence microscopy after hormone treatments as a preliminary indication of potential changes in the cell type-specific expression of the different aquaporins.

**Fig 5:**
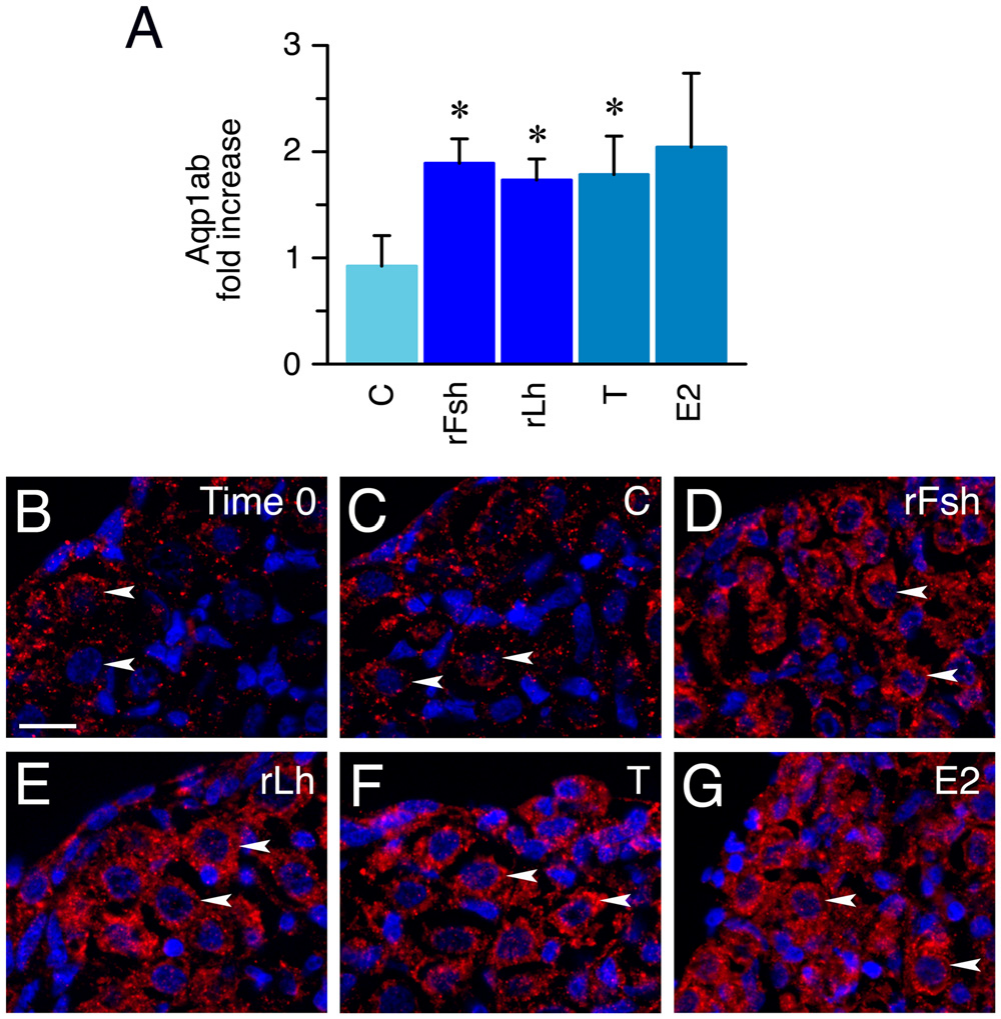
Effect of gonadotropins and steroid hormones on Aqp1ab protein synthesis in explants at the resting stage *in vitro*. (A) Fold increase (mean ± S.E.M; n = 3 replicates) in the relative amount of Aqp1ab protein in explants treated with 100 ng/ml of rFsh or rLh, or 10 ng/ml of T or E2. *, P < 0.05 with respect the control group. See S2 Fig for the individual immunoblots. (B-G) Representative Aqp1ab immunostaining in explants treated as above. Arrowheads point to the spermatogonia. Reactions were visualized as in Fig 2. Scale bar, 20 μm. Images from sections at different spermatogenic stage or after hormone treatments were taken with the same fluorescence intensity and exposure time than those used for the controls.

Due to the small size of the testis explants at the resting period, IP using the Aqp1ab antibody was carried out prior to the immunoblot to concentrate the target protein (S2 Fig). These experiments confirmed that both gonadotropins and T incremented the total amount of phosphorylated and dephosphorylated Aqp1ab in the testis (Fig 5A and S2 Fig), while the immunostaining indicated that the Aqp1ab polypeptides accumulated mainly in spermatogonia (Fig 5B–5G). The effect of E2 on Aqp1ab protein abundance was not significant due to the large variation of the intensity of the Aqp1ab immunoreactive bands in the immunoblots (Fig 5A and S2 Fig), although a more intense Aqp1ab staining was seen in the spermatogonia from estrogen-treated explants (Fig 5G).

**Fig 6:**
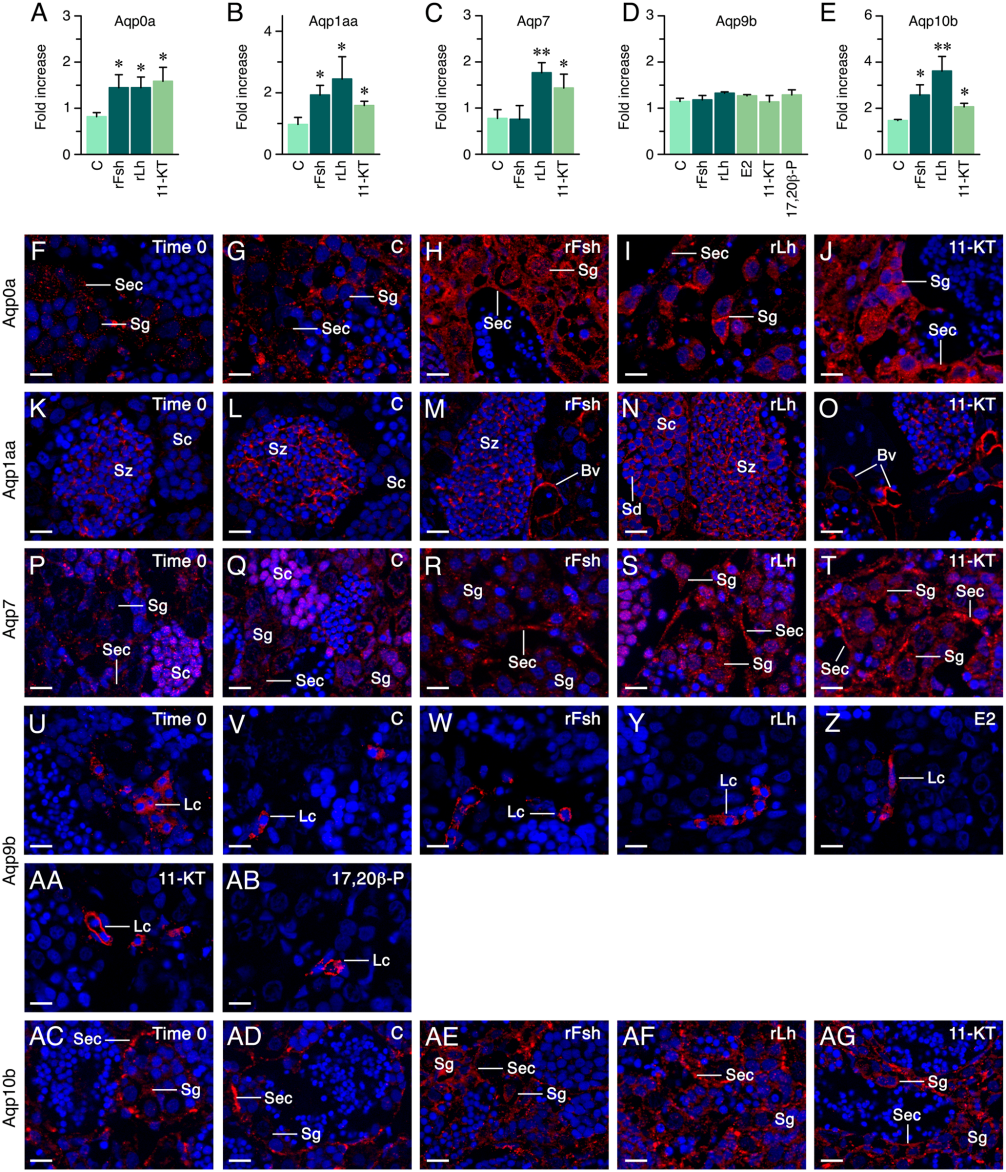
Effect of gonadotropins and steroid hormones on aquaporin protein synthesis in explants at the spermatogenic stage *in vitro*. (A-E) Fold increase (mean ± S.E.M; n = 3 replicates) in the relative amount of Aqp0a, -1aa, -7, -9b and -10b protein in explants treated with 100 ng/ml of rFsh or rLh, or 10 ng/ml of E2, 11-KT or 17,20β-P. **, P < 0.01; *, P < 0.05 with respect the control group. See S3 Fig for the individual immunoblots. (F-AG) Representative immunostaining of explants treated as above using the corresponding antibodies. Reactions were visualized as in Fig 2. Scale bar, 10 μm. Images from sections at different spermatogenic stage or after hormone treatments were taken with the same fluorescence intensity and exposure time than those used for the controls. Sec, Sertoli cell; Lc, Leydig cell; Sg, spermatogonia; Sc, spermatocyte; Sd, spermatid; Sz, spermatozoa; Bv, blood vessel.

In explants at the spermatogenic stage, semiquantitation of aquaporin expression by Western blot showed significantly higher levels of Aqp0a, -1aa and -10b after treatment with rFsh and rLh, and of Aqp7 after rLh exposure, while the translation of all these aquaporins was also upregulated with 11-KT although to a different extent (Fig 6A–6C and 6E, and S3 Fig). These data thus agree with the qRT-PCR data. For Aqp9b, however, the total protein levels did not change after gonadotropin or steroid treatments (Fig 6D and 6U–6AB), despite that each of these hormones, except T, upregulated the corresponding transcripts in the testis. Immunostaining of treated explants revealed a more intense Aqp0a signal in Sertoli cells (Fig 6H) and spermatogonia (Fig 6I) after rFsh and rLh exposure, respectively, while 11-KT was apparently increasing Aqp0a immunoreacton in both cell types (Fig 6J). In contrast, rFsh seemed to enhance Aqp1aa staining in the endothelia of intratesticular blood vessels (Fig 6M), despite that this gonadotropin was not found to significantly increase *aqp1aa* expression in the testis, whereas rLh apparently elevated Aqp1aa immunostaining in spermatocytes (Fig 6N). The androgen 11-KT had the same effect that rFsh on Aqp1aa in blood vessels, but had no apparent effect on the protein expression of the channel in germ cells (Fig 6O). The rLh and 11-KT appeared to increase the translation of Aqp7 in spermatogonia (Fig 6S and 6T), whereas both hormones, and apparently also rFsh, incremented the Aqp7 signals in the basal region of the Sertoli cells (Fig 6R–6T). Both gonadotropins and 11-KT also enchanced Aqp10b immunostaining in spermatogonia at this stage, whereas the signal in Sertoli cells was unchanged (Fig 6AE–6AG).

**Fig 7:**
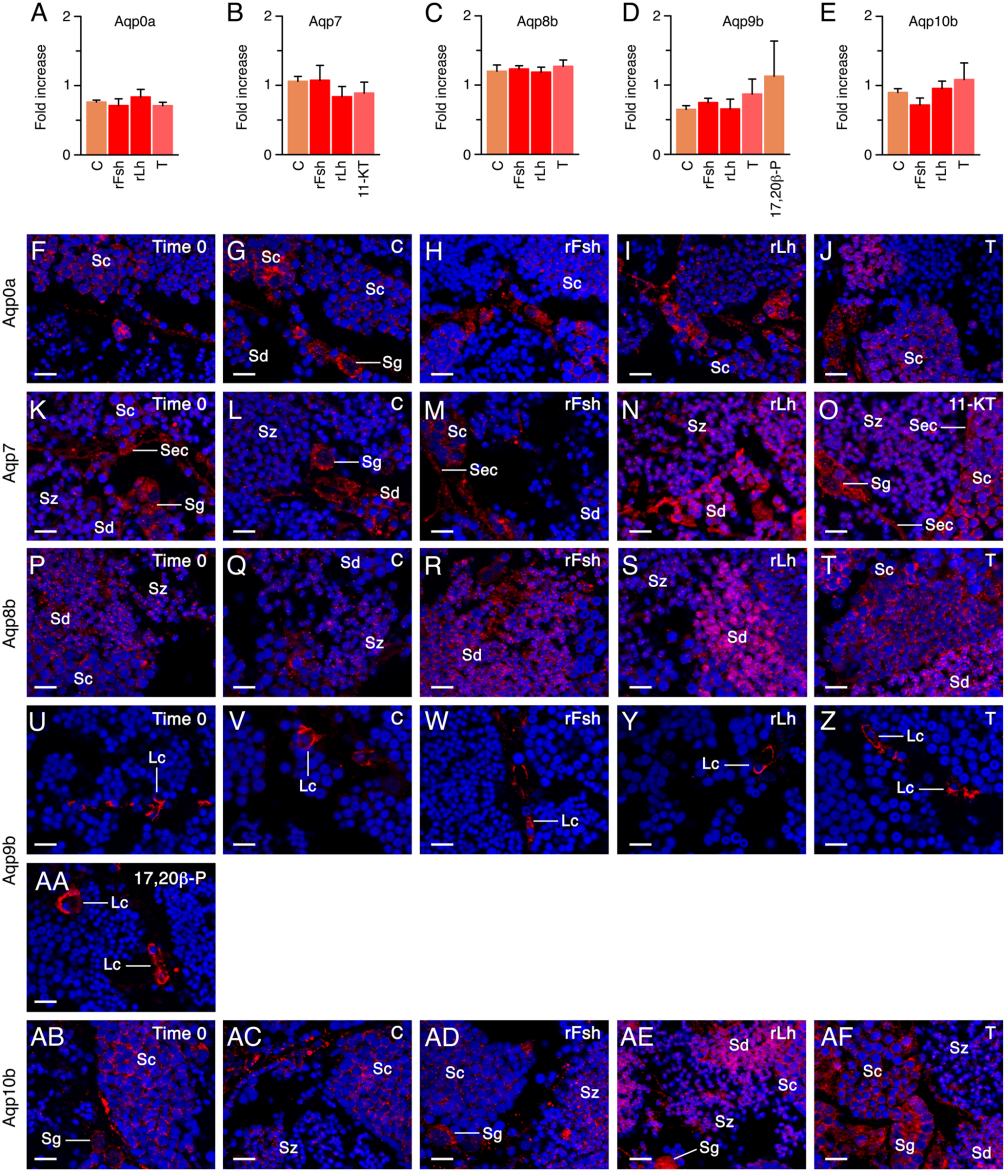
Effect of gonadotropins and steroid hormones on aquaporin protein synthesis in explants at the spermiation stage *in vitro*. (A-E) Fold increase (mean ± S.E.M; n = 3 males) in the relative amount of Aqp0a, -7, -8b, -9b and -10b protein in explants treated with 100 ng/ml of rFsh or rLh, or 10 ng/ml of T, 11-KT or 17,20β-P. **, P < 0.01; *, P < 0.05 with respect the control group. See S4 Fig for the individual immunoblots. (F-AF) Representative immunostaining of explants treated as above using the corresponding antibodies. Reactions were visualized as in Fig 2. Scale bar, 10 μm. Images from sections at different spermatogenic stage or after hormone treatments were taken with the same fluorescence intensity and exposure time than those used for the controls. Sec, Sertoli cell; Lc, Leydig cell; Sg, spermatogonia; Sc, spermatocyte; Sd, spermatid; Sz, spermatozoa; Bv, blood vessel.

During the spermiation period, immunoblot analyses did not show significantly different levels of the total amounts of Aqp0a, -7, -8b, -9b and -10b polypeptides in explants treated with gonadotropins or steroids (Fig 7A–7E and S4 Fig), even though the corresponding transcripts were upregulated by these hormones. However, immunofluorescence microscopy of the explants revealed that T enhanced Aqp0a staining in spermatocytes (Fig 7J), whereas Aqp7 positive signals in Sertoli cells, spermatocytes and spermatids (Fig 7N and 7O), and specifically those for Aqp8b in spermatids (Fig 7S and 7T), were stronger after rLh and 11-KT stimulation. Similarly, rLh and T increased Aqp10b signals in spermatogonia, spermatocytes and spermatids (Fig 7AE and 7AF). Finally, as observed during the spermatogenic period, no differences in Aqp9b staining in Leydig cells were detected after the hormone treatments (Fig 7U–7AA).

## Discussion

**Fig 8:**
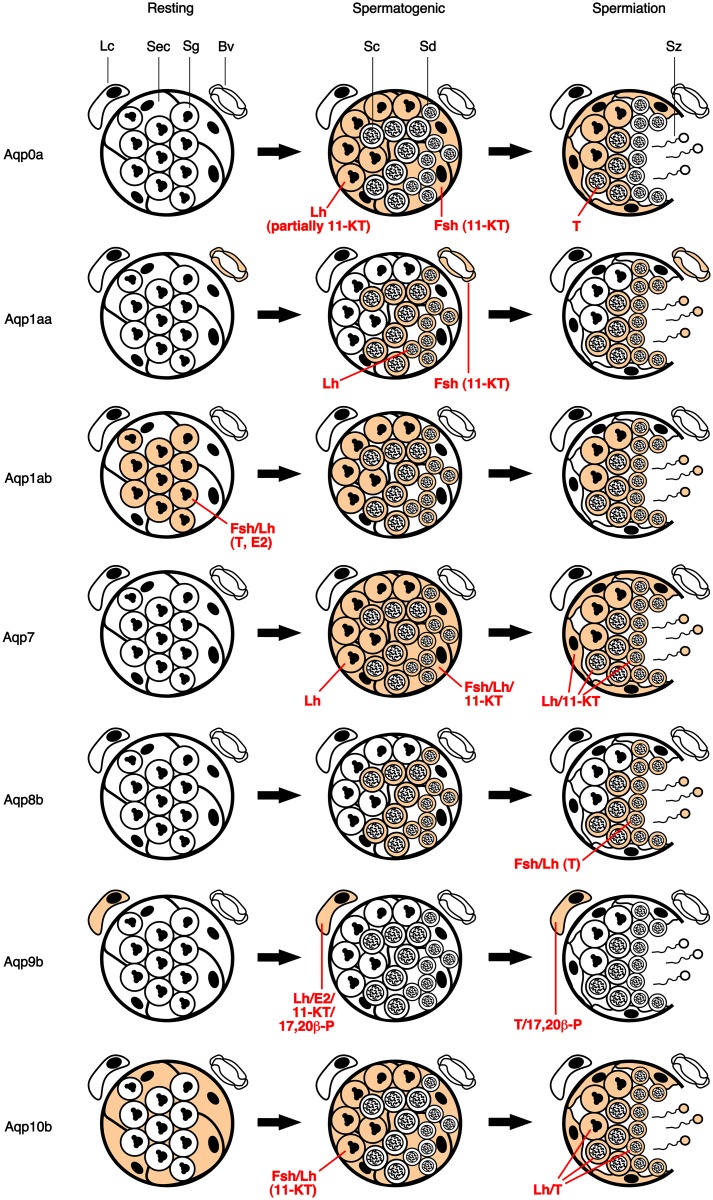
Schematic representation of the expression and endrocrine regulation of testicular aquaporins during seabream spermatogenesis. Colored cell types at each stage indicate aquaporin expression demonstrated by immunofluorescence microscopy. Hormones involved in aquaporin mRNA and/or protein regulation are in red letters. Whether the Fsh and Lh regulation is mediated by steroids is indicated in parenthesis. Sec, Sertoli cell; Lc, Leydig cell; Sg, spermatogonia; Sc, spermatocyte; Sd, spermatid; Sz, spermatozoa; Bv, blood vessel.

The present study reveals a complex pattern of cell-type specific expression and endocrine regulation of seven different aquaporin paralogs, Aqp0a, -1aa, -1ab, -7, -8b, -9b and -10b, during spermatogenesis of a marine teleost. The combined data sets allowed us to propose the first model of the differential short-term regulation of testicular aquaporins by gonadotropins and sex steroids in a non mammalian vertebrate (Fig 8). This model is based on the corroborative results of aquaporin mRNA and protein expression levels, except during the spermiation stage, during which presumably high endogenous expression of most of the aquaporins investigated likely masked the effects observed in the *in vitro* studies.

### Aquaporins in testicular somatic cells and blood vessels endothelia

In mammals, it has been proposed that different aquaporins present in Sertoli cells, such as AQP0, -4, and AQP8-10, may play an important role for the absorption/secretion of the seminiferous tubular fluid, which provides the necessary nutritional and hormonal environment for spermatogenesis, as well as for the progression of sperm from the testis to the epididymis [6]. In this study, we found that seabream Sertoli cells also express several aquaporins, Aqp0a, -7 and -10b; however, while Aqp10b is already detected in Sertoli cells before spermatogenesis, Aqp0 and -7 expression is activated specifically when germ cells enter into meiosis (Fig 8). In our previous study, expression of Aqp7 and -10b in Sertoli cells was not detected [28], possibly because this study was carried out on spermiating testes only, where the strong immunoreaction for these two aquaporins in germ cells could mask additional positive reaction in the underlying Sertoli cells.

Before and during spermatogenesis, Aqp10b in Sertoli cells was not regulated *in vitro* by gonadotropins, androgens, estrogen or progestin, which may suggest that short-term *in vitro* cultures were not long enough to detect the effect of these hormones, or that Aqp10b in Sertoli cells may be regulated by other signals such as growth factors [35, 36]. In contrast, when plasma levels of T and 11-KT increase during spermatogenesis, Aqp0a in Sertoli cells was upregulated by 11-KT produced in response to rFsh, which was likely mediated by the stimulation of Fsh cognate receptors in the steroidogenic Leydig cells [33, 37], which in turn can activate androgen nuclear receptors expressed in Sertoli cells [38, 39, 40]. For Aqp7, however, no changes in the testicular transcript or protein level were recorded after rFsh stimulation during spermatogenesis, although an increased accumulation of the channel at the basal region of Sertoli cells apparently occurred at this stage after rFsh exposure. By contrast, during spermiation the *aqp7* transcript levels in the testis and Aqp7 immunostaining in Sertoli cells were upregulated by rLh, possibly through the local production of 11-KT, since this androgen alone elicited the same effects even though the activation by rLh was not sensitive to TRIL. These observations could therefore suggest the regulation of Aqp7 intracellular trafficking in Sertoli cells by direct Fsh stimulation during spermatogenesis, and the Lh-triggered activation of the expression of this channel specifically during spermiation. Although these potential mechanisms remain to be demonstrated, the specific positive regulation of Aqp0a, -7 and -10b in seabream Sertoli cells may indicate a role of these channels in the movement of water and solutes across the seminiferous epithelium during germ cell development as well as during spermiation, when an increase of the tubular fluid and the hydration of the seminal fluid occurs [21]. However, in mammals AQP0 and -4 have been suggested to mediate membrane junctions [41, 42], and therefore Aqp0a channels might also contribute to cell adhesion structures in seabream Sertoli cells that are part of the testis-blood barrier, which in teleosts is generally formed during the transition from spermatocytes to early spermatids, when tight junctions are established between Sertoli cells [43].

In seabream Leydig cells, only Aqp9b was found to be expressed throughout the spermatogenic cycle, and this channel appeared to be Leydig cell-specific since it was not detected in any other cell type of the testis. This finding is in contrast to that reported in mammals, where Leydig cells seem to express AQP0 and the tetrapod-specific AQP2 and -5, in addition to AQP9, which is also found in germ cells [3, 19, 20, 44, 45]. In seabream, testicular *aqp9b* mRNA levels, but not those of the protein product, were upregulated upon rLh, E2, 11-KT and 17,20β-P treatment at the spermatogenic stage, while during spermiation *aqp9b* was upregulated by T and 17,20β-P (Fig 8). The positive effect on *aqp9b* expression by rLh was not dependant on androgens, suggesting the presence of Lh regulated pathways in Leydig cells controlling *aqp9b* transcription. However, the observation that 11-KT, E2 and 17,20β-P alone could upregulate *aqp9b* expression suggest that autocrine and/or paracrine mechanisms, through steroidogenic enzymes expressed in germ cells [40, 46], may also exist. The existence of these mechanisms is supported by the presence of androgen and estrogen nuclear receptors in fish Leydig cells [38, 40, 47, 48], and by the reported effect of androgens and estrogens on Leydig cell ultrastructure and/or steroidogenesis [49, 50], previously observed in teleosts. The activation of *aqp9b* transcription in seabream Leydig cells by the progestin 17,20β-P is more intriguing, since expression of the progestin nuclear receptor in these cells has been reported in zebrafish (*Danio rerio*) [51] but not in other teleosts [40, 52, 53], although membrane-bound progestin receptors [54] may be involved, as it might occur in rat Leydig cells [55]. However, despite that *aqp9b* in seabream Leydig cells is transcriptionally regulated by gonadotropins and steroids during spermatogenesis, the role of this channel is unclear, although it may be involved in the transport of water and non-charged solutes in these interstitial cells, as suggested for mammals [56, 57].

In addition to the Leydig cells, the only cells in the seabream testicular interstitial tissue showing aquaporin expression were the endothelial cells of blood vessels close to the seminiferous tubules, which were found to express Aqp1aa throughout the spermatogenic cycle (Fig 8). The presence of Aqp1aa in blood vessels may contribute to the transcellular water transport from the blood stream to the seminiferous tubules. This hypothesis would be consistent with the increased Aqp1aa translation by Fsh and 11-KT that seemed to occur during spermatogenesis. Although this mechanism is unclear because no changes in testicular *aqp1aa* transcription were also noted after rFsh stimulation, it may agree with the expression of the Fsh and androgen receptors in mammalian endothelial cells [58, 59].

### Aquaporins in germ cells

During seabream germ cell development, the first aquaporin to be expressed in proliferating spermatogonia was Aqp1ab, whereas activation of Aqp0a, -7 and -10b was associated with the increased testicular secretion of 11-KT and the entry into meiosis of diploid cells, and that of Aqp1aa and -8b was specific of haploid cells (Fig 8). Such tight developmental regulation of aquaporins suggest the role of these channels for fluid efflux during the decrease of cell volume occuring during the series of cell divisions from spermatogonia to spermatids and spermatozoa [60], and for the transport of nutrient molecules from Sertoli cells that may be required during cell differentiation. However, aquaporins expressed in germ cells, except Aqp0a, are prevalent in mature spermatozoa [28], and therefore the coordinated synthesis and storage of these channels in intracellular vesicles during spermatogenesis can be an additional mechanism involved.

The regulation of the different aquaporin paralogs in seabream germ cells was different during the spermatogenic cycle, and both steroid-dependent and -independent mechanisms were observed (Fig 8). In spermatogonia at the resting stage, Aqp1ab was the only channel expressed and was upregulated by gonadotropins through T production, although the stimulation of *aqp1ab* expression by E2 was stronger than that of T suggesting that the effect of T could be mediated by the rapid aromatization of the androgen in Leydig cells [46]. However, at this stage we noted that both gonadotropins, E2, and T, induced spermatogonia proliferation *in vitro*, according to the known role of estrogens in spermatogonial renewal through the activation of estrogen receptors in Sertoli cells [37, 61], and therefore whether androgens and/or estrogens directly or indirectly stimulate *aqp1ab* transcription in spermatogonia remains to be addressed. In any event, the early expression of Aqp1ab during seabream spermatogenesis is very similar to the developmental control of this paralog during oogenesis, where Aqp1ab is synthesized already in meiosis-arrested primary growth (previtellogenic) oocytes and later transported to the oocyte plasma membrane where it mediates oocyte hydration associated with meiosis reinitiation [25, 30, 62, 63]. However, Aqp1ab synthesis in seabream early oocytes is activated by Fsh-triggered progestins [32, 63], whereas spermatogonial *aqp1ab* was apparently not affected by short-term exposure to 17,20β-P. These observations may suggest a different endrocrine control of *aqp1ab* in male and female germ cells, but experiments with longer incubation times of seabream spermatogonia in the presence of progestins would be necessary to confirm this hypothesis.

During the progression of spermatogenesis, spermatogonia start to express Aqp0a, -7 and -10b (Fig 8), together with Aqp1ab. However, while expression of Aqp1ab, -7 and -10b is maintained in differentiating spermatocytes and spermatids, and in spermatozoa through the spermiation period, that of Aqp0a remains only up to the spermatocyte stage. In contrast, Aqp1aa and -8b expression was detected only in spermatocytes and spermatids, as well as in spermatozoa during spermiation, suggesting that these channels were upregulated late in germ development possibly for a later role during the activation and maintenance of sperm motility [64, 65]. Transcription of Aqp1ab was not regulated by gonadotropins or sex steroids during spermatogenesis or spermiation, and thus this channel appears to be stored throughout germ cell differentiation as it occurs during oogenesis [62, 63]. The qRT-PCR and immunostaining data suggested however that spermatogonial Aqp0a and -10b were upregulated by 11-KT produced in response to Fsh and/or Lh during spermatogenesis, whereas Aqp8b in spermatids was activated by gonadotropin-triggered T only during spermiation (Fig 8). These findings indicate that most aquaporins in seabream germ cells are controlled by androgen-dependent mechanisms, through androgen receptors expressed either in Sertoli cells or in the germ cells themselves [40, 66] as reported for mammals [67, 68]. The upregulation of Aqp0a and -10b in spermatogonia by 11-KT may be concomitant with the entry into meiosis of these cells, since in several teleosts it has been shown that 11-KT stimulates spermatogonial proliferation toward meiosis [37]. In our study, we did not observe significantly increased PCNA-based spermatogonia proliferation by 11-KT in any of the spermatogenic stages investigated, possibly because longer times of incubation with the androgen are needed to elicit an effect [69].

Our data also indicate, however, that some aquaporins expressed in seabream germ cells, such as Aqp1aa, -7, and partially Aqp0a, can be regulated by Lh through steroid-independent mechanisms. This finding would agree with the recently reported presence of the Lh receptor in seabream spermatocytes and spermatids [70]. In the flatfish Senegalese sole (*Solea senegalensis*), it has been shown that ligand-activated Lh receptor in spermatids released into the tubular lumen directs the expression of genes involved in spermiogenesis [70]. Therefore, our observations suggest that Aqp1aa and -7 in germ cells could be under direct Lh regulation as part of the transcriptional reprogramming process occurring during the transformation of haploid spermatids into a highly specialized, motile and fertile spermatozoa [8]. However, we also noted that during spermiation, T or 11-KT alone can upregulate Aqp0a, -1aa, -7 and -10b expression in germ cells (Fig 8), suggesting that androgens might also be involved in the control of seabream spermiogenesis, as reported for mammals [71], as well as regulating aquaporin expression during this process.

## Conclusions

The results of the present study confirm the presence of a complex aquaporin network in the testis of a teleost fish, and indicate that the expression of the different aquaporin paralogs in somatic and germ cells during spermatogenesis is differentially regulated by Fsh and Lh through androgen-dependent and independent mechanisms. These findings emphasize the potential importance of aquaporin-mediated water and solute transport for the control of the local fluid balance during teleost germ cell development. Our data suggest that androgens are the major steroids regulating aquaporin expression in the seabream testis, although the specific molecular mechanisms involved should be investigated in further studies. Also, the regulatory pathways for the aquaporins showing dual expression in Sertoli and germ cells, such as Aqp0a, -7 and -10b, need to be confirmed in the future using isolated cell preparations. Nevertheless, the present work provides the first model of aquaporin regulation during the spermatogenesis of a non mammalian vertebrate as a step forward towards the elucidation of the physiological roles of these important channels in the reproductive systems.

## Supporting Information

S1 Fig Localization of proliferative germ cells after hormone treatments *in vitro*. (A-H) Representative PCNA immunostaining of testis explants at the resting stage after stimulation with 100 ng/ml of rFsh or rLh, or hormone vehicle (control; C), or 10 ng/ml of steroid hormones (T, E2, 11-KT and 17,20β-P), for 24 h. (I) Quantification of percent PCNA-positive germ cells in explants at the resting, spermatogenic and spermiation stages. Data (mean ± S.E.M.) are from six testicular samples and represent the results of three separate experiments on three different pools of male fish. *, P < 0.05; **, P < 0.01, with respect the control group at time zero.
(TIF)

S2 Fig Western blot analysis of the effects of gonadotropins and steroid hormones on Aqp1ab protein synthesis in explants at the resting stage *in vitro*. (A) Representative immunoprecipitation (IP) experiment on testis extracts using rabbit IgG or the seabream Aqp1ab antibody showing the specific precipitation of Aqp1ab. (B) Aqp1ab immunoblots of testis explants before (Time 0) and after treatment with 100 ng/ml of rFsh or rLh, 10 ng/ml of T or E2, or hormone vehicle (control; C), and immunoprecipitated with Aqp1ab. The three blots are technical replicates. The arrows indicate Aqp1ab monomer and the arrowheads phosphorylated Aqp1ab. The IgG heavy chain (IgG-HC) is indicated. Molecular mass markers are on the left.
(TIF)

S3 Fig Western blot analysis of the effects of gonadotropins and steroid hormones on aquaporin protein synthesis in explants at the spermatogenic stage *in vitro*. (A) Immunoblots for Aqp0a, -1aa, -7, -9b or -10b of testis explants before (Time 0) and after treatment with 100 ng/ml of rFsh or rLh, 10 ng/ml of E2, 11-KT or 17,20β-P, or hormone vehicle (control; C). Alpha-tubulin (Tuba) was used as loading control. The three blots are technical replicates. The arrows indicate aquaporin monomers whereas the arrowheads indicate potential posttranslational modifications. Molecular mass markers are on the left.
(TIF)

S4 Fig Western blot analysis of the effects of gonadotropins and steroid hormones on aquaporin protein synthesis in explants at the spermiation stage *in vitro*. (A) Immunoblots for Aqp0a, -7, -8b, -9b and -10b of testis explants before (Time 0) and after treatment with 100 ng/ml of rFsh or rLh, 10 ng/ml of T, 11-KT or 17,20β-P, or hormone vehicle (control; C). Alpha-tubulin (Tuba) was used as loading control. The three blots correspond to three different fish. The arrows indicate aquaporin monomers whereas the arrowheads indicate potential posttranslational modifications. Molecular mass markers are on the left.
(TIF)

S1 Table Primer sequences used for qRT-PCR. (PDF)
