# Supplementary figures and images for: Gonadotropin-Activated Androgen-Dependent and Independent Pathways Regulate Aquaporin Expression during Teleost (Sparus aurata) Spermatogenesis
Source: PLoS One. 2015 Nov 17;10(11):e0142512. doi: 10.1371/journal.pone.0142512 (PMC4648546; doi:10.1371/journal.pone.0142512)

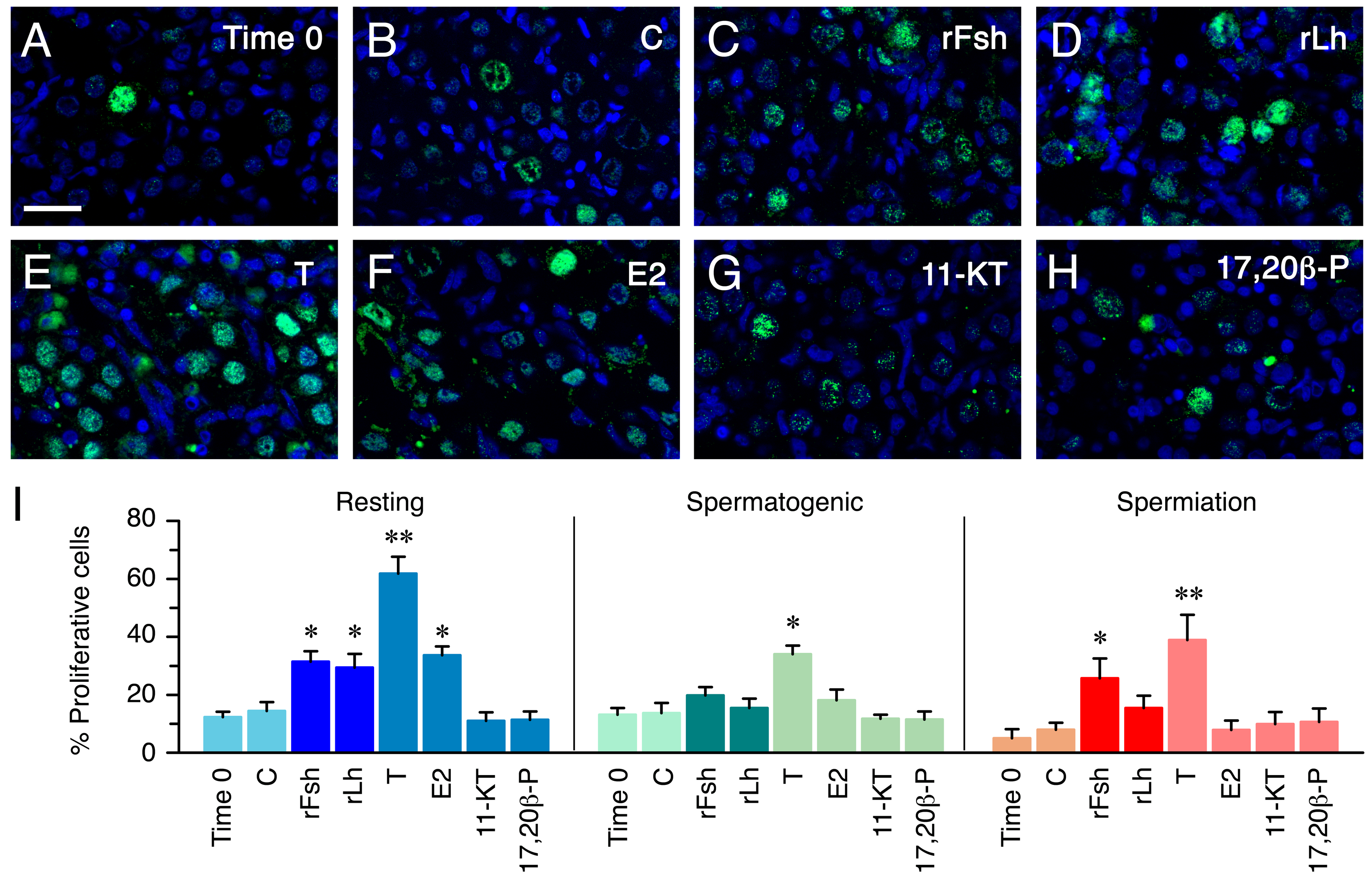

Supplement: S1 Fig — (A-H) Representative PCNA immunostaining of testis explants at the resting stage after stimulation with 100 ng/ml of rFsh or rLh, or hormone vehicle (control; C), or 10 ng/ml of steroid hormones (T, E2, 11-KT and 17,20β-P), for 24 h. (I) Quantification of percent PCNA-positive germ cells in explants at the resting, spermatogenic and spermiation stages. Data (mean ± S.E.M.) are from six testicular samples and represent the results of three separate experiments on three different pools of male fish. *, P < 0.05; **, P < 0.01, with respect the control group at time zero. (TIF) [file pone.0142512.s001.tif]

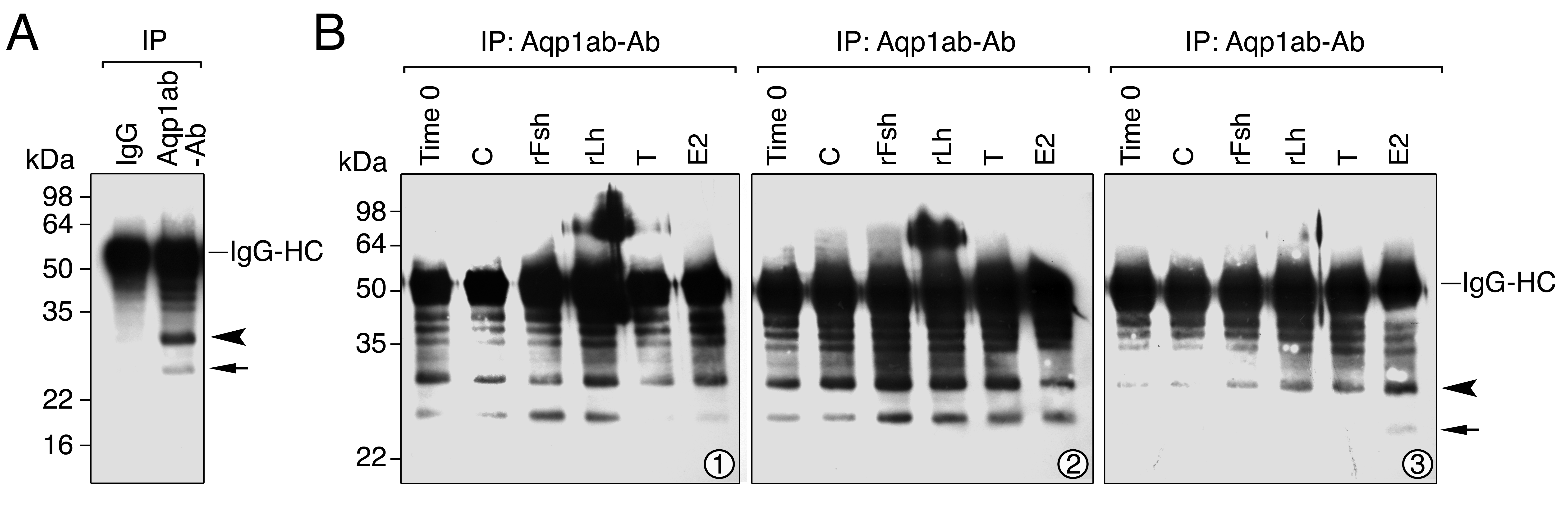

Supplement: S2 Fig — (A) Representative immunoprecipitation (IP) experiment on testis extracts using rabbit IgG or the seabream Aqp1ab antibody showing the specific precipitation of Aqp1ab. (B) Aqp1ab immunoblots of testis explants before (Time 0) and after treatment with 100 ng/ml of rFsh or rLh, 10 ng/ml of T or E2, or hormone vehicle (control; C), and immunoprecipitated with Aqp1ab. The three blots are technical replicates. The arrows indicate Aqp1ab monomer and the arrowheads phosphorylated Aqp1ab. The IgG heavy chain (IgG-HC) is indicated. Molecular mass markers are on the left. (TIF) [file pone.0142512.s002.tif]

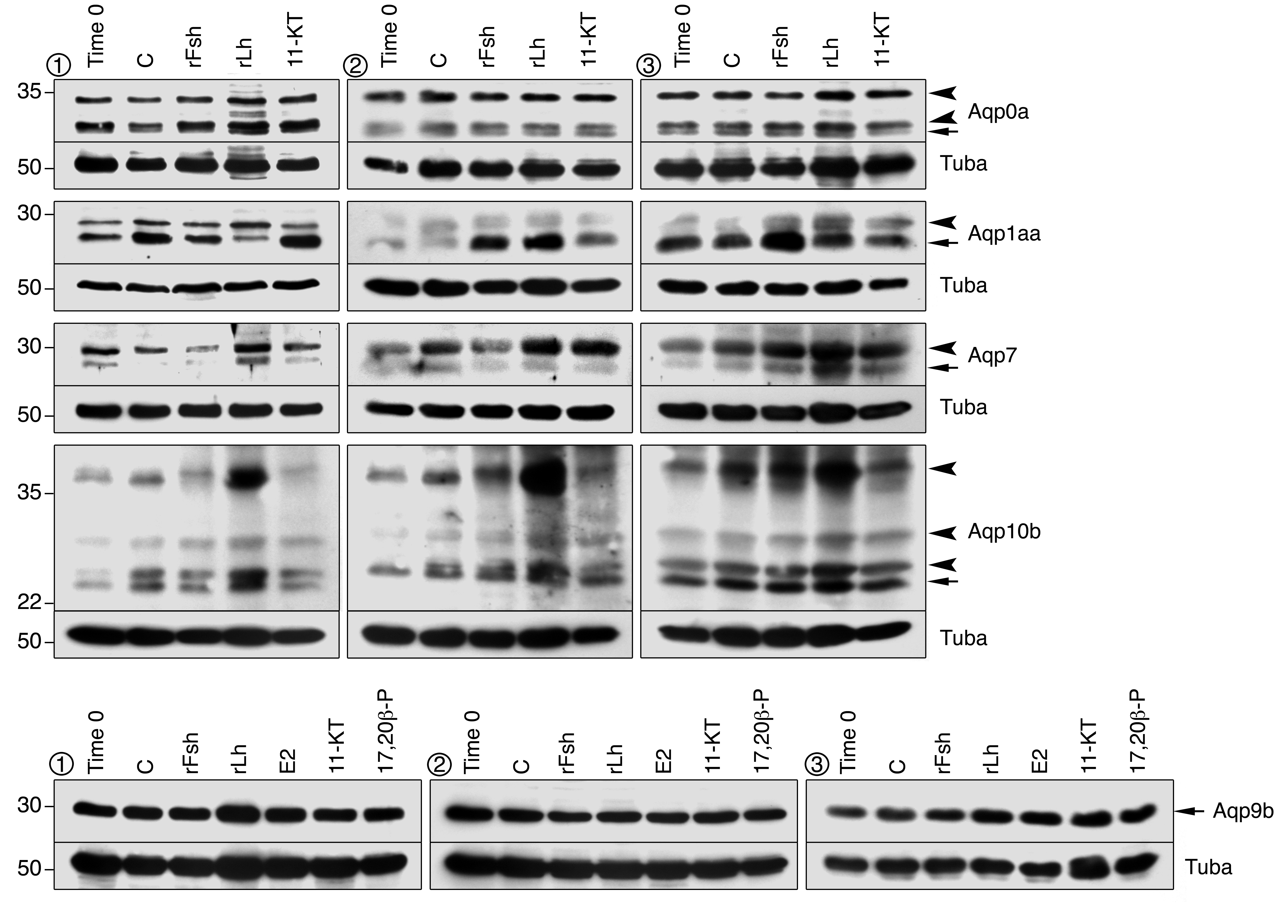

Supplement: S3 Fig — (A) Immunoblots for Aqp0a, -1aa, -7, -9b or -10b of testis explants before (Time 0) and after treatment with 100 ng/ml of rFsh or rLh, 10 ng/ml of E2, 11-KT or 17,20β-P, or hormone vehicle (control; C). Alpha-tubulin (Tuba) was used as loading control. The three blots are technical replicates. The arrows indicate aquaporin monomers whereas the arrowheads indicate potential posttranslational modifications. Molecular mass markers are on the left. (TIF) [file pone.0142512.s003.tif]

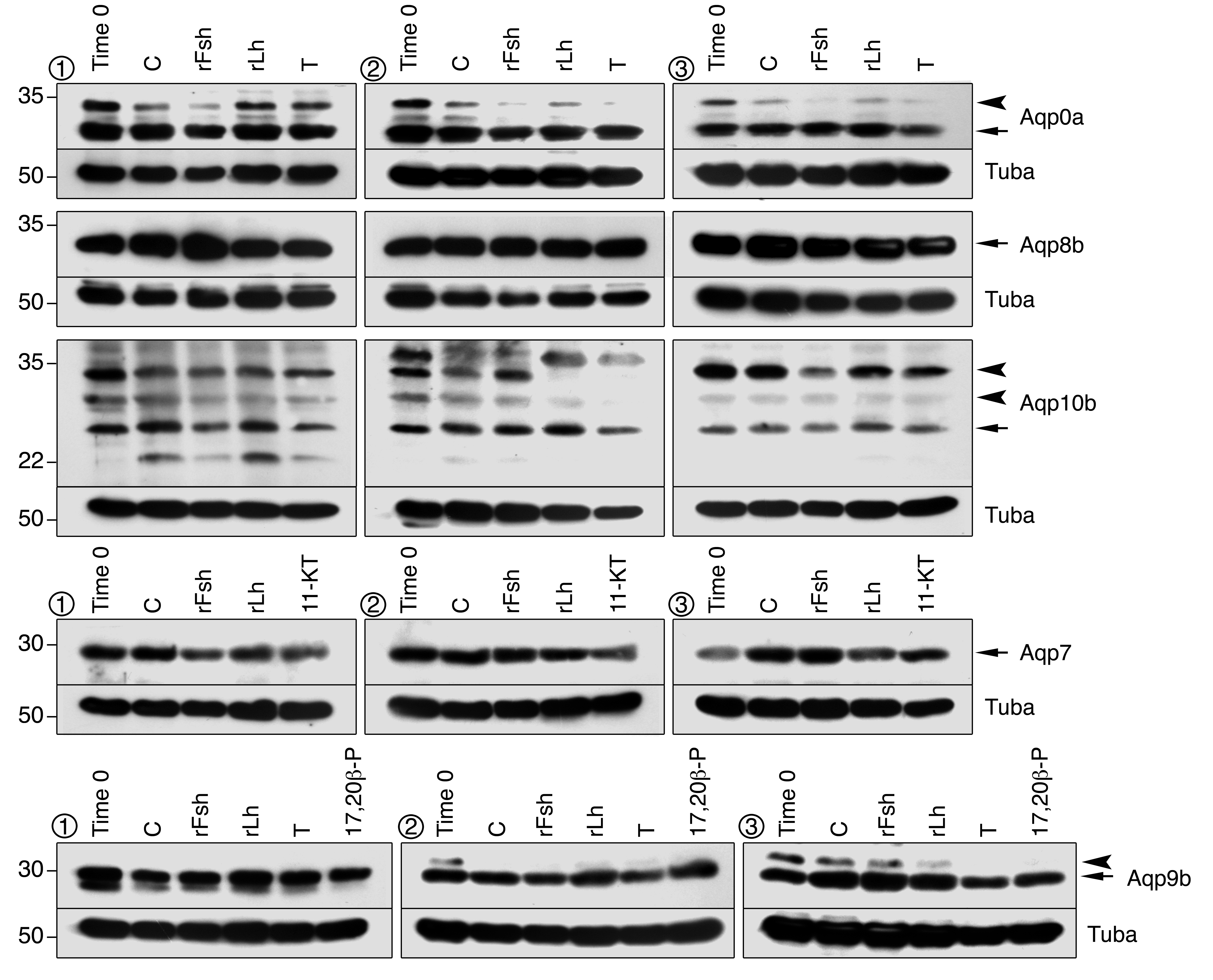

Supplement: S4 Fig — (A) Immunoblots for Aqp0a, -7, -8b, -9b and -10b of testis explants before (Time 0) and after treatment with 100 ng/ml of rFsh or rLh, 10 ng/ml of T, 11-KT or 17,20β-P, or hormone vehicle (control; C). Alpha-tubulin (Tuba) was used as loading control. The three blots correspond to three different fish. The arrows indicate aquaporin monomers whereas the arrowheads indicate potential posttranslational modifications. Molecular mass markers are on the left. (TIF) [file pone.0142512.s004.tif]
