# Supplementary material for: Gonadotropin-Activated Androgen-Dependent and Independent Pathways Regulate Aquaporin Expression during Teleost (Sparus aurata) Spermatogenesis
Source: PLoS One. 2015 Nov 17;10(11):e0142512. doi: 10.1371/journal.pone.0142512 (PMC4648546; doi:10.1371/journal.pone.0142512)
Supplement: S1 Table — (PDF) [file pone.0142512.s005.pdf]

S1 Table. Primer sequences used for qRT-PCR

Gene	GenBank #	Forward/Reverse	Amplicon (bp)	Efficiency
aqp0a	KC589385	CAGGGGCCTCTCTCTCTCTT/ GGAAAAGCATGCAGAAGTCC	118	2.11
aqp1aa	AY626939	TCCTGAACAATTTCACGAACC/ GCCTCCGTTAACGTCGTAGT	171	1.89
aqp1ab	AY626938	GCGACGGAGTGATGTCAAAGG/ AGATAAGAGCCGCCGCTATGC	203	1.89
aqp7	KC589386	TGAAGGACTTGGTGCAGTCA/ CCTAGGCCAAACACCATCAT	148	2.01
aqp8b	DQ889225	TCCGGCTTTAGTGAGCAACT/ TCCTGTCTCCAAGCAGGAGT	110	2.11
aqp9b	KC589387	ATCCTGGCCATCACTGACA/ GGTTGATCGGATAGCCACAG	124	1.99
aqp10b	AY363261	GTGCTGGTAATCGGCGTCT/ GGCCTTGAAAACATCCACTC	120	1.96
rps18	AY587263	ACTAAGAACGGCCATGCACCACCAC/ GAATTGACGGAAGGGCACCACC	149	1.96

All primers are flanking at least one intron (based on zebrafish genomic organization).
